# Hypothesis: *Cryptosporidium* genetic diversity mirrors national disease notification rate

**DOI:** 10.1186/s13071-015-0921-3

**Published:** 2015-06-06

**Authors:** Katsuhisa Takumi, Simone M. Cacciò, Joke van der Giessen, Lihua Xiao, Hein Sprong

**Affiliations:** Centre for Infectious Disease Control, National Institute for Public Health and the Environment (RIVM), P.O. Box 1, Bilthoven, 3720 The Netherlands; Department of Infectious, Parasitic and Immunomediated Diseases, Istituto Superiore di Sanità, Rome, Italy; Division of Foodborne, Waterborne and Environmental Diseases, National Center for Emerging and Zoonotic Infectious Diseases, Centers for Disease Control and Prevention, Atlanta, USA

**Keywords:** *Cryptosporidium hominis*, *Cryptosporidium parvum*, GP60, Population genetics, Molecular epidemiology

## Abstract

**Background:**

Cryptosporidiosis is a gastrointestinal disease affecting many people worldwide. Disease incidence is often unknown and surveillance of human cryptosporidiosis is installed in only a handful of developed countries. A genetic marker that mirrors disease incidence is potentially a powerful tool for monitoring the two primary human infected species of *Cryptosporidium.*

**Methods:**

We used the molecular epidemiological database with *Cryptosporidium* isolates from ZoopNet, which currently contains more than 1400 records with their sampling nations, and the names of the host species from which the isolates were obtained. Based on 296 *C. hominis* and 195 *C. parvum* GP60 sequences from human origin, the genetic diversities of *Cryptosporidium* was estimated for several nations. Notified cases of human cryptosporidiosis were collected from statistics databases for only four nations.

**Results:**

Genetic diversities of *C. hominis* were estimated in 10 nations in 5 continents, and that of *C. parvum* of human origin were estimated in 15 nations. Correlation with reported incidence of human cryptosporidiosis in four nations (the Netherlands, United States, United Kingdom and Australia) was positive and significant. A linear model for testing the relationship between the genetic diversity and incidence produced a significantly positive estimate for the slope (*P*-value < 0.05).

**Conclusions:**

The hypothesis that genetic diversity at GP60 locus mirrors notification rates of human cryptosporidiosis was not rejected based on the data presented. Genetic diversity of *C. hominis* and *C. parvum* may therefore be an independent and complementary measure for quantifying disease incidence, for which only a moderate number of stool samples from each nation are sufficient data input.

## Background

*Cryptosporidium* is one of the most common diarrhea-causing parasitic genera in the world [1-3]. Human cryptosporidiosis is a notifiable disease in several, mainly European countries [4] where cryptosporidiosis cases are primarily recorded in reports of incidental water and food borne outbreaks [5].

*Cryptosporidium* parasites that are important for humans are classified into the two main species, *C. hominis* and *C. parvum,* and further into subtype families based on sequence variability in the GP60 gene [6, 7] that encodes a surface glycoprotein. The gene might be under selection by host antibody response. Global variability within and between species was investigated based on its amino-acid polymorphism [8]. *Cryptosporidium* is a parasite that has a sexual lifecycle, with most of the stages of the lifecycle being haploid while the zygote is diploid and the oocyst contains four sporozoites that should represent two different sets of identical recombinant genes if parental isolates are genetically different. This means that sexual recombination is likely to be common if strains from different genetic heritages infect the same animal or person. Mallon *et al.* argued that the populations of *C. parvum* in the UK were panmictic, based on genetic diversity whereas those of *C. hominis* are more clonal [9]. *Cryptosporidium* isolates from humans and from animals in various countries have been sequenced and compiled into a database [10, 11]. This database, made possible by a European consortium (ZoopNet), is a source of molecular and epidemiological information about the protozoan infection in humans and in animals (*Giardia* and *Cryptosporidium*). In particular, sampled countries cover parts of the globe extending to the continents of Asia and Africa in addition to America, Europe, and Australia. The broad geographic range is a strong asset of the database, if we can infer the population risk of human cryptosporidiosis from the regional sequence data.

Here, we test the hypothesis that the genetic diversity of *Cryptosporidium* at the GP60 locus from human isolates positively correlates with the population risk. In other words, the exposure to infectious oocysts in a human population could be indirectly measured by quantifying the genetic diversities of *C. hominis* and *C. parvum* DNA sequences, as found in human fecal samples. Each cell division is a potential event for a novel genetic variant. Hence, a higher number of cell divisions per unit time should drive genome to a greater diversity. The rate of cell divisions for *Cryptosporidium* species that infect humans could be quantified using an annual incidence rate as a proxy. We refer by genetic diversity to an estimate for the population size based on the coalescent theory in molecular evolution literature [12]. A similar approach was previously applied to Lyme disease risk in the Netherlands [13], where it revealed a concomitant increase in the incidence of Lyme disease in humans and in the genetic diversity of *Borrelia burgdorferi* sensu lato in ticks. It presents a precedent case validating the application of molecular evolution approach to estimating the extent to which humans are exposed to the infectious microorganism, where no alternative approach is possible.

For the current study, national public health statistics and the existing *Cryptosporidium* sequence database are empirical input into the molecular evolutionary analysis to test our hypothesis.

## Methods

### Notification rates of human cryptosporidiosis per nation

Notification rates of human cryptosporidiosis were obtained from the literature (summarized in Table 1). For our analysis, we required the reported notification rates to be compatible with the sequence sampling years. In addition, we required that the rates are comparable among nations. This meant that a nation’s notification rate should be expressed and reported as estimated number of cases per 100,000 residents per year (incidence). Countries which reported rates in other numerical terms, e.g., cases per 1,000,000, were not excluded. Data in which epidemiology of human cryptosporidiosis was quantified in a cross-sectional study (e.g., fraction of positive stools in a sample population) were not included in the further analysis, due to the lack of a simple conversion between study-outcomes.

**Table 1:** Notified cases of human cryptosporidiosis per 100,000 inhabitants

Nation	Reporting period (total years)	Notified cases (mean ± s.d.)	References
Netherlands	2003 – 2005 (3)	1.8 ± 1.2	[16]
US	2006 – 2008 (3)	3.2 ± 0.9	[17]
UK	2006 – 2008 (3)	7.1 ± 1.1	[4]
Australia	2004 – 2010 (7)	12.9 ± 5.0	[18, 19]

### Cryptosporidium spp. database

A network of European public health and veterinary institutions (ZoopNet) was established to generate and collect molecular epidemiological data from *Giardia* and *Cryptosporidium* isolates. The current database (February 2013) contains more than 1400 records of GP60 sequences, sampled countries, and host species from which isolates were collected. *Cryptosporidium* isolates of human (n = 882), animal (n = 374) or unknown origin (n = 199) present in the ZoopNet-database [11] were collected from European countries on a voluntary basis. *Cryptosporidium* GP60 DNA sequences were also downloaded and frequently updated from the Entrez Nucleotide Database (GenBank) at the National Center for Biotechnology Information and processed as described [8]. The GP60 sequences were downloaded together with the geographical origin (country) and the host species from which the isolate originated. Sequences which did not originate from natural isolates were excluded. Sequences that were too short to cover regions of variation were also excluded from further analysis. Share of GenBank GP60 sequences in the ZoopNet database is approximately 75 %. All molecular epidemiological data for 296 *C. hominis* and 195 *C. parvum* sequences were stored and analysed in Bionumerics (Version 6.6, Applied Math, Belgium). DNA sequences and data used for this study are available upon request.

Subtype families of the isolates were identified based on amino acid sequence similarity. *Cryptosporidium* sequences of known species and subtype families were retrieved from GenBank using the accession numbers AY738184 to AY738196 [7]. For each human isolate, Smith Waterman Similarity scores were calculated on the pair of amino acid sequences using BLOSUM62 rule and Gap penalty of 4.

### Estimation of *Cryptosporidium* genetic diversity per nation

Genetic diversity of human isolates was estimated separately for *C. hominis* and *C. parvum*. For each species, GP60 sequences were grouped per nation and the sequences were aligned using the multiple alignment software MAFFT [14]. An observed base substitution in the alignment should have same contribution to the estimation of genetic diversity whether it is due to point mutation, viral infection or recombination. Subsequently, the alignments were analyzed per nation by a molecular evolution approach using the software Bayesian Evolutionary Analysis by Sampling Trees (BEAST, version 1.8) [12]. Reconstructing phylogenetic tree is not our primary scope. Hence, we used the default setting in the software: Hasegawa-Kishino-Yamamoto (HKY) model of DNA evolution and a constant mutation rate for each site of the GP60 sequences. We ran each simulation for one million updates and discarded 10 % burn-in. At least two GP60 sequences from human isolates per nation were necessary for this analysis, and nations were excluded from the analysis when this condition was not met. BEAST produced a list of posterior samples for the population size estimates based on the coalescent theory.

### Statistical analysis

Notification rates account for the two major *Cryptosporidium* species. Hence, we combined the genetic diversities estimated separately for each species into one measure. We performed all statistical computations described in this section using the software Mathematica, version 9 (Champaign, IL, Wolfram Research, Inc).

**Fig. 1:**
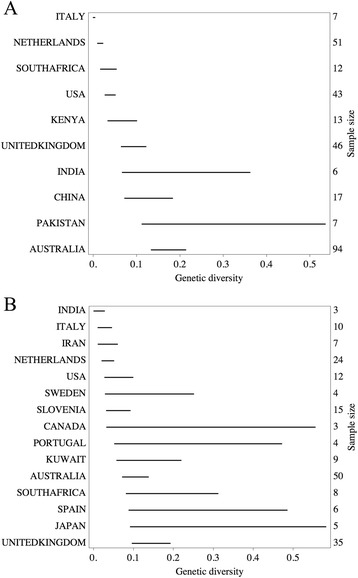
Genetic diversity of two human-infective species of *Cryptosporidium. C. hominis* (panel **a**) and *C. parvum* of human origin (panel **b**). The horizontal axis represents the genetic diversity of GP60 sequences per nation: a coalescent effective population size multiplied by mutation rate per generation at GP60 locus. The horizontal line aligned to a nation name represents the posterior credible interval around the estimated genetic diversity. The integer next to the right vertical axis is the number of GP60 sequences used in the estimation

Overall genetic diversity was obtained by summing the two posterior distributions from BEAST runs, weighted according to the observed frequencies of *C. hominis* and *C. parvum* in the total number of isolates per nation (Fig. 1a and 1b). To calculate the weighted sum, we applied the standard kernel-smoothing function in Mathematica to the list of posterior samples to obtain a continuous distribution.

The relationship between the genetic diversity and the notification rate from the same nation was obtained by calculating the bivariate, i.e., 2-dimensional, distribution from the univariate distribution on genetic diversity and the univariate distribution on annual notification rate. The two random variables were assumed independent. To calculate the distribution on the notification rate, we applied the kernel-smoothing function to the list of annual notification rates reported in national statistics.

We calculated Pearson correlation and Spearman rank, together with their significance values based on 20 random samples from the relationship describing the genetic diversity and the notification rate. The relationship was calculated by summing nation-specific bivariate distributions, weighted according to the observed numbers of *Cryptosporidium* sequences per nation. The numbers of sampled sequences in each country are listed in Fig. 1.

In addition to the correlation analysis, we tested whether the mean notification rate is proportional to the mean genetic diversity. We applied a generalized linear model with Gaussian response and identity link to the set of four pairs of mean values: the genetic diversity and the notification rate. The goodness of fit of the linear model was assessed by the likelihood ratio test. *P*-values smaller than 0.05 were considered to be significant.

## Results

Genetic diversities of *C. hominis* were estimated for ten nations from five continents (Fig. 1a) by a molecular evolution approach [12]. The analysis of a total of 296 GP60 sequences revealed that genetic diversity estimates differed among nations. The highest genetic diversity was estimated from 94 GP60 sequences from Australia, whereas the lowest genetic diversity was estimated from seven GP60 sequences from Italy. Estimates of genetic diversities were independent of the sampling sizes, but tend to be uncertain with smaller sampling sizes (Fig. 1a).

Genetic diversities of *C. parvum* from human isolates were estimated for 15 nations (Fig. 1b). The highest genetic diversity was estimated from 35 GP60 sequences from the UK. The lowest genetic diversity was estimated from three GP60 sequences from India. As for *C. hominis*, the estimates of genetic diversities were independent of the sampling sizes, but tend to be less certain with smaller sampling sizes (Fig. 1b).

**Fig. 2:**
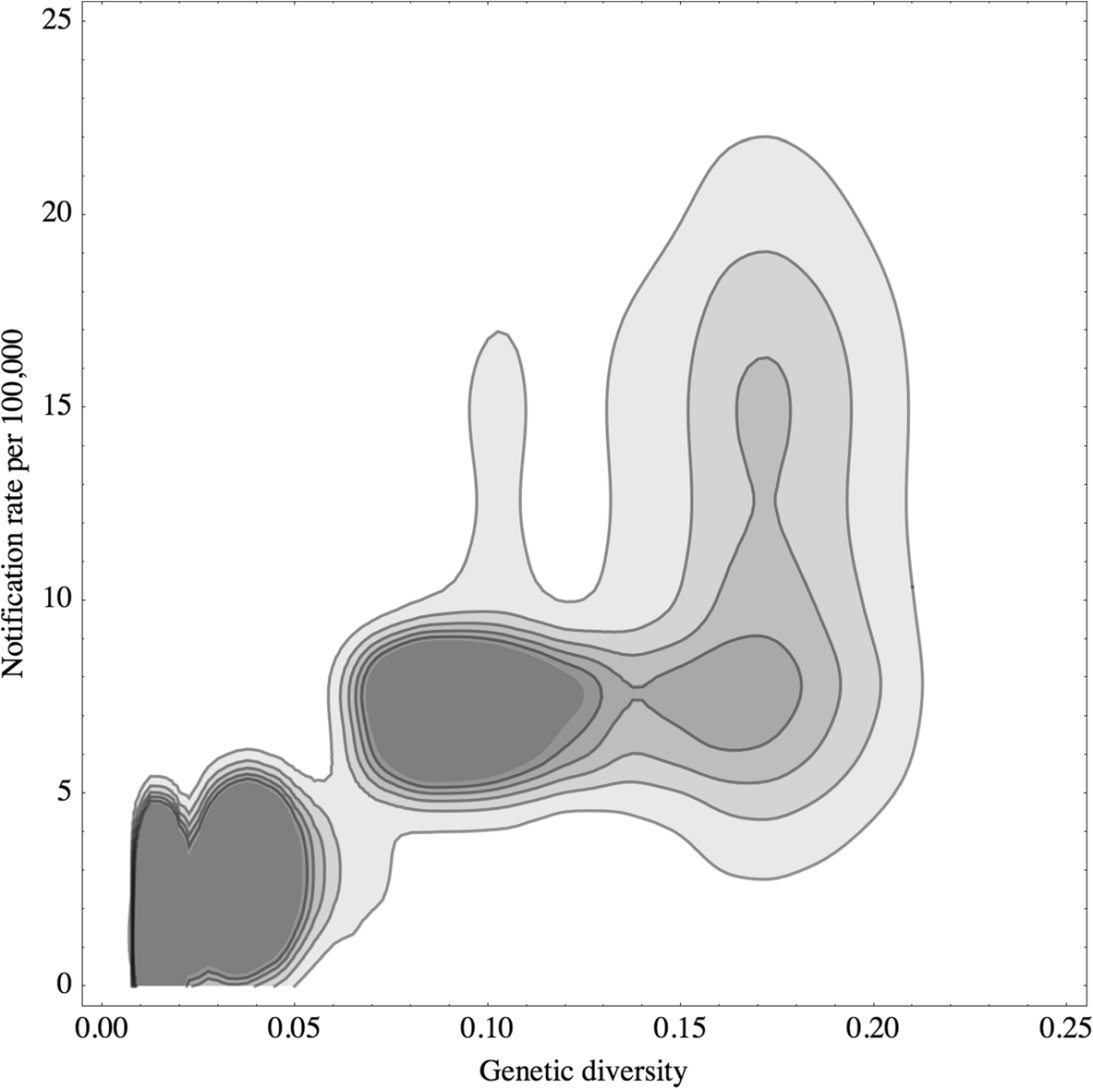
Distribution of *Cryptosporidium* genetic diversities and notification rates. A pair of estimates regarding the notification rate and the genetic diversity is colored in gray shade according to its probability density. A darker shade indicates a higher probability density. A closed line connects the points at which probability density is equal to a certain value. Estimates are based on the data from four countries: The Netherlands, USA, UK, and Australia

Notification rates from public health statistics (Table 1) varied between nations. The mean notification rate was estimated to be 1.8 cases per 100,000 residents in the Netherlands, 3.2 cases in the US, 7.1 cases in the UK, and 12.9 cases in Australia (Table 1). We were unable to collect similar epidemiological data from other nations where the protozoan genetic diversity could be estimated from the sequence database alone (Fig. 1a and 1b). The lack of epidemiological data therefore reduced the number of nations available for a correlation analysis. Four nations (The Netherlands, USA, UK, and Australia) met the minimal data requirement to quantify the relationship between the genetic diversity and the notification rate, that is to say, two DNA sequences per species and two annual incidence estimates. Nonetheless, we observed that the higher the estimated genetic diversity, the higher the national notification rates (Fig. 2).

Pearson correlation between the *Cryptosporidium* genetic diversity and the annual notification rates was positive (0.73) and significant (*P*-value < 0.05). Spearman rank was positive (0.8) and significant (*P*-value < 0.05).

**Fig. 3:**
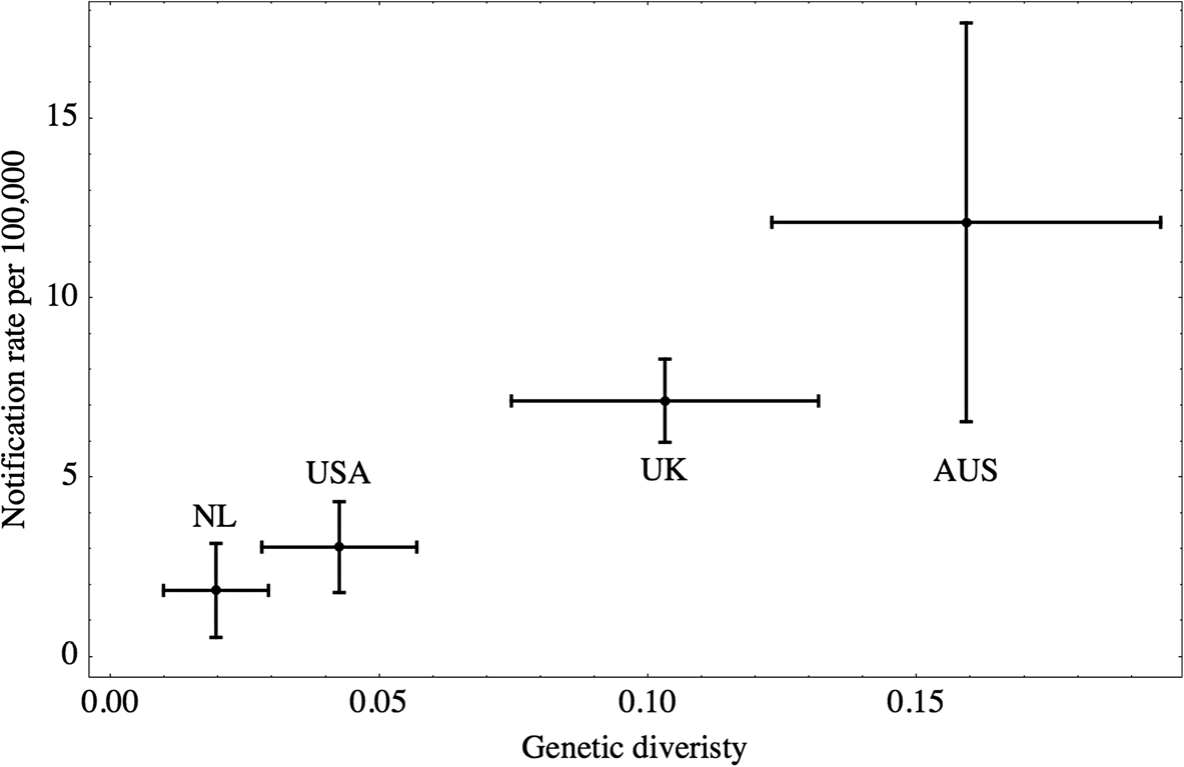
Mean and standard deviation of *Cryptosporidium* genetic diversities and notification rates in four nations. A cross is positioned at the estimated mean values and displayed in the size of one standard deviation in each direction of the axes. Genetic diversities were estimated based on *C. hominis* and *C. parvum* only originating from humans. Capital letters are abbreviations for nation names: the Netherlands (NL), the United States (USA), the United Kingdom (UK), and Australia (AUS)

An independent analysis of the datasets from the four nations based upon a generalized linear model resulted in a significant linear relationship (*P*-value < 0.05) between the estimated mean genetic diversity and the estimated mean notification rate (Fig. 3).

The relationship between estimated genetic diversity and the notification rates could be specific only to the combination of *C. hominis* and *C. parvum* genetic diversities. To exclude the possibility that a significant association with the notification rates would become absent without combining the two species, we applied the statistical analyses using the *C. hominis* dataset alone. Spearman rank was positive (0.8) and significant (*P*-value < 0.05). The slope of the generalized linear model was significantly greater than zero (*P*-value < 0.05). We repeated the additional analyses using the *C. parvum* dataset alone. Spearman rank was not significant (*P*-value = 0.1). The slope of the generalized linear model was not significantly different from zero (*P*-value = 0.2).

## Discussion

A study made a suggested observation that genetic variation at GP60 locus might be essential for the parasite’s long-term success [15]. It is therefore meritorious to target GP60 for analyzing genetic and epidemiological associations of the infectious disease. In this study, we demonstrated that genetic diversities of GP60 sequences from human isolates correlate positively with national notification rates of human cryptosporidiosis. The mechanism underlying this positive correlation would require a careful examination of many biological, environmental, and social factors that influence the transmission and the epidemiology of the infection. It was beyond the scope of this study to address this complex issue.

Nonetheless, we like to comment on some factors that might affect the result of this study. First, some *C. parvum* from human isolates might directly originate from one or more animal reservoirs. Extensive zoonotic transmission would change the estimated genetic diversity in either upward direction or downward direction, depending on the genetic diversity of zoonotic isolates in animal reservoirs. Hence, one might expect only a weak relationship between estimated genetic diversities with human cryptosporidiosis notification rates, when extensive transmission from animal reservoirs would occur. A significant and positive slope of the linear model indicated however, that zoonotic transmission interfered little on the relationship between the estimated genetic diversity and the epidemiology in those countries. Second, some infections might have been acquired abroad. Over-represented, foreign acquired infection is expected to level the estimated genetic diversities between nations. Contrary to this expectation, the slope of the linear model testing the relationship between the genetic diversity and incidence was positive and significant, in the developed nations where the residents might travel more frequently compared to those in developing nations, indicating that the overall *Cryptosporidium* genetic diversity was predominantly determined by autochthonous cases. It is possible that, due to social and environmental circumstances different from the nations analyzed in this study, human cryptosporidiosis acquired from livestock animals or from abroad alters the genetic diversity and its relationship to epidemiology in those nations.

In the molecular evolutionary approach that we have adopted, genetic diversity is proportional to the product of the mutation rate and the estimate for population size based on the coalescent theory [12]. Thus, a relative difference in the genetic diversity of *Cryptosporidium* could reflect either a relative difference in the estimates for the *Cryptosporidium* population sizes between nations, or a relative difference in mutation rate. We postulate that mutation rate is a less-likely source of variation and by eliminating the possibility, we opt for the idea that the *Cryptosporidium* population size reflects the national notification rate of human cryptosporidiosis.

Random sampling is an assumption in the molecular evolution approach, and selectively sampled sequences are expected to influence the estimate on genetic diversity. One might expect for example that two isolates collected from an outbreak in a single site are more closely related than e.g., two isolates collected in two distant provinces in China. This sampling bias might change the estimates on genetic diversity in each nation in terms of absolute value. However, we expect such bias to influence independently the reported disease incidence in corresponding nations, and therefore, aligning genetic diversity and disease incidence in a number of nations by a spurious mechanism such as sampling bias appears unlikely.

In theory, our hypothesis could be tested using any genetic elements, including MLST and microsatellite. GP60 sequence is rich in repeats of tri-nucleotides (TCA, TCG and TCT). The length of the tri-nucleotide repeats can change by a deletion and insertion events, and this process is not accounted for by a model of DNA evolution by a point mutation in the molecular evolutionary approach [12]. The duplication process would affect the estimates for the population size based on the coalescent theory to an unknown extent. Resolution to detect correlation would depend on the rate of evolutionary changes of the genetic elements, relative to a typical time scale between one infection to the next. Even so, the more pronounced limitations of our study would be the following: the nations used to validate the relationship between the genetic diversity and disease notification rates is a small subset of all nations. There is no consensus on a globally representative group of nations and how large the group should be.

A longitudinal study in a single nation is an additional test case to our hypothesis regarding the population exposure and the population risk. Seasonal incidence and dated molecular sequences from those affected in the same season in the same nation are empirical input to test this hypothesis based upon independent observations.

## Conclusions

Hypothesis that *Cryptosporidium* genetic diversity mirrors the notification rates of human cryptosporidiosis could not be rejected based on the datasets from the four developed countries. This inference from the molecular sequences may be an independent and alternative approach to estimate the risk of human cryptosporidiosis where collection of the samples is an economically suitable option.
